# Routine screening for pulmonary embolism in COVID-19 patients at the emergency department: impact of D-dimer testing followed by CTPA

**DOI:** 10.1007/s11239-021-02508-1

**Published:** 2021-06-23

**Authors:** Daniël A. Korevaar, Ilayda Aydemir, Maartje W. Minnema, Kaoutar Azijli, Ludo F. Beenen, Jarom Heijmans, Nick van Es, Mohanad al Masoudi, Lilian J. Meijboom, Saskia Middeldorp, Prabath W. Nanayakkara, Rick I. Meijer, Peter I. Bonta, Josien van Es

**Affiliations:** 1 Department of Respiratory Medicine, Amsterdam UMC, Meibergdreef 9, 1105 AZ Amsterdam, The Netherlands; 2 Department of Respiratory Medicine, Onze Lieve Vrouwe Gasthuis, Amsterdam, The Netherlands; 3 Section Emergency Medicine, Emergency Department, Amsterdam Cardiovascular Sciences, Amsterdam Public Health Research Institute, Amsterdam UMC, Amsterdam, The Netherlands; 4 Department of Radiology and Nuclear Medicine, Amsterdam Cardiovascular Sciences, Amsterdam UMC, Amsterdam, The Netherlands; 5 Section General and Acute Internal Medicine, Department of Internal Medicine, Amsterdam Cardiovascular Sciences, Amsterdam Public Health Research Institute, Amsterdam UMC, Amsterdam, The Netherlands; 6 Department of Vascular Medicine, Amsterdam Cardiovascular Sciences, Amsterdam UMC, Amsterdam, The Netherlands; 7 Department of Internal Medicine and Radboud Institute of Health Sciences, Radboud UMC, Nijmegen, The Netherlands

**Keywords:** COVID-19, Pulmonary embolism, D-dimer, Computed tomography angiography, Sensitivity and specificity

## Abstract

COVID-19 patients have increased risk of pulmonary embolism (PE), but symptoms of both conditions overlap. Because screening algorithms for PE in COVID-19 patients are currently lacking, PE might be underdiagnosed. We evaluated a screening algorithm in which all patients presenting to the ED with suspected or confirmed COVID-19 routinely undergo D-dimer testing, followed by CT pulmonary angiography (CTPA) if D-dimer is ≥ 1.00 mg/L. Consecutive adult patients presenting to the ED of two university hospitals in Amsterdam, The Netherlands, between 01-10-2020 and 31-12-2020, who had a final diagnosis of COVID-19, were retrospectively included. D-dimer and CTPA results were obtained. Of 541 patients with a final diagnosis of COVID-19 presenting to the ED, 25 (4.6%) were excluded because D-dimer was missing, and 71 (13.1%) because they used anticoagulation therapy. Of 445 included patients, 185 (41.6%; 95%CI 37.0–46.3) had a D-dimer ≥ 1.00 mg/L. CTPA was performed in 169 of them, which showed PE in 26 (15.4%; 95%CI 10.3–21.7), resulting in an overall detection rate of 5.8% (95%CI 3.9–8.4) in the complete study group. In patients with and without PE at CTPA, median D-dimer was 9.84 (IQR 3.90–29.38) and 1.64 (IQR 1.17–3.01), respectively (p < 0.001). PE prevalence increased with increasing D-dimer, ranging from 1.2% (95%CI 0.0–6.4) if D-dimer was 1.00–1.99 mg/L, to 48.6% (95%CI 31.4–66.0) if D-dimer was ≥ 5.00 mg/L. In conclusion, by applying this screening algorithm, PE was identified in a considerable proportion of COVID-19 patients. Prospective management studies should assess if this algorithm safely rules-out PE if D-dimer is < 1.00 mg/L.

## Highlights

- Pulmonary embolism is prevalent in COVID-19 patients but symptoms of both conditions overlap, resulting in diagnostic challenges in the emergency department
- A screening algorithm was evaluated, in which COVID-19 patients presenting to the emergency department underwent routine D-dimer testing, if ≥ 1.00 mg/L followed by CTPA
- 41.6% of patients had a D-dimer ≥ 1.00 mg/L, and CTPA showed pulmonary embolism in 15.4% of them, resulting in an overall detection rate of 5.8%
- Using this screening algorithm, pulmonary embolism was identified in a considerable proportion of COVID-19 patients, which has important therapeutic consequences

## Introduction

Patients with COVID-19 are at increased risk of developing pulmonary embolism (PE) [1–4]. However, symptoms of COVID-19 and PE overlap, resulting in diagnostic challenges in patients presenting to the emergency department (ED) [5, 6]. CT pulmonary angiography (CTPA) is considered the gold standard for PE, but performing this test in all COVID-19 patients would be logistically challenging, and lead to considerable costs and unnecessary exposure to radiation and contrast agents. It is unknown to which extent diagnostic algorithms for ruling-out PE developed in the non-COVID-19 population, such as those incorporating the YEARS criteria or the Wells’ rule [7–11], are efficient and safe for PE screening in COVID-19 patients [12]. Furthermore, these algorithms rely on D-dimer testing for ruling-out PE, but D-dimer is often increased in COVID-19 patients, regardless the presence of PE [13].

Because screening algorithms for PE in COVID-19 patients are currently lacking, PE might be underdiagnosed in this population, which would result in suboptimal treatment. In this study, we evaluated a pragmatic screening algorithm in which all COVID-19 patients presenting to the ED routinely undergo D-dimer testing, followed by CTPA if D-dimer is ≥ 1.00 mg/L, regardless of the pre-test probability of PE. Our primary aims were to assess (1) the proportion of COVID-19 patients with a D-dimer ≥ 1.00 mg/L, (2) the prevalence of PE at CTPA in those with a D-dimer ≥ 1.00 mg/L, and (3) D-dimer results in those with and without PE at CTPA.

## Methods

In the Amsterdam University Medical Centers, The Netherlands, a pragmatic screening algorithm for PE was implemented in the fall of 2020, and has been routinely applied in all patients presenting to the ED with suspected or confirmed COVID-19. According to this algorithm, all patients with suspected or confirmed COVID-19 are considered suspected for PE, and D-dimer testing is routinely performed. If D-dimer is ≥ 1.00 mg/L, CTPA is subsequently done.

In the current retrospective analysis, consecutive patients aged 18 years or older who presented to the ED of one of the two hospital locations (Academic Medical Center or VU University Medical Center) between 01 October 2020 and 31 December 2020, with a final diagnosis of COVID-19 were included. Patients were eligible regardless of the severity of respiratory symptoms and pre-test probability of PE. A final diagnosis of COVID-19 was defined as either confirmed COVID-19 upon ED presentation (i.e. a positive RT-PCR for SARS-CoV-2 infection prior to ED presentation), or suspected COVID-19 upon ED presentation (defined as fever without other cause, cough, dyspnea, flulike symptoms, or loss of smell or taste) that was subsequently confirmed (i.e. a positive RT-PCR during ED presentation or, if applicable, within 7 days after hospital admission). Patients without a confirmed SARS-CoV-2 infection were not eligible, because the initial level of COVID-19 suspicion varied considerably across these patients. We excluded patients who were on anticoagulation therapy, and those in whom the local protocol was violated and no D-dimer testing had been performed during ED presentation.

D-dimer and CTPA results were extracted from medical records. D-dimer was measured using the Innovance D-Dimer Assay (Siemens Healthcare Diagnostics, Marburg, Germany). CTPAs were assessed by attending radiologists with varying levels of experience as part of clinical practice, not blinded to clinical information or D-dimer results. In the Academic Medical Center, CTPA was performed on a dual source scanner (Somatom Force, Siemens Healthineers, Erlangen, Germany). In the VU Medical Center, this was a single source scanner (GE Discovery 750 HD, GE Healthcare, Milwaukee, WI, USA).

Data were summarized descriptively. Proportions were reported with 95% confidence intervals (95%CI) calculated with the Clopper-Pearson method. Medians were compared using Mann–Whitney U-test. The study protocol was assessed by our institute’s medical ethics committee and exempted from ethical approval, as the Medical Research Involving Human Subjects Act does not apply to this retrospective observational study.

## Results

**Fig. 1:**
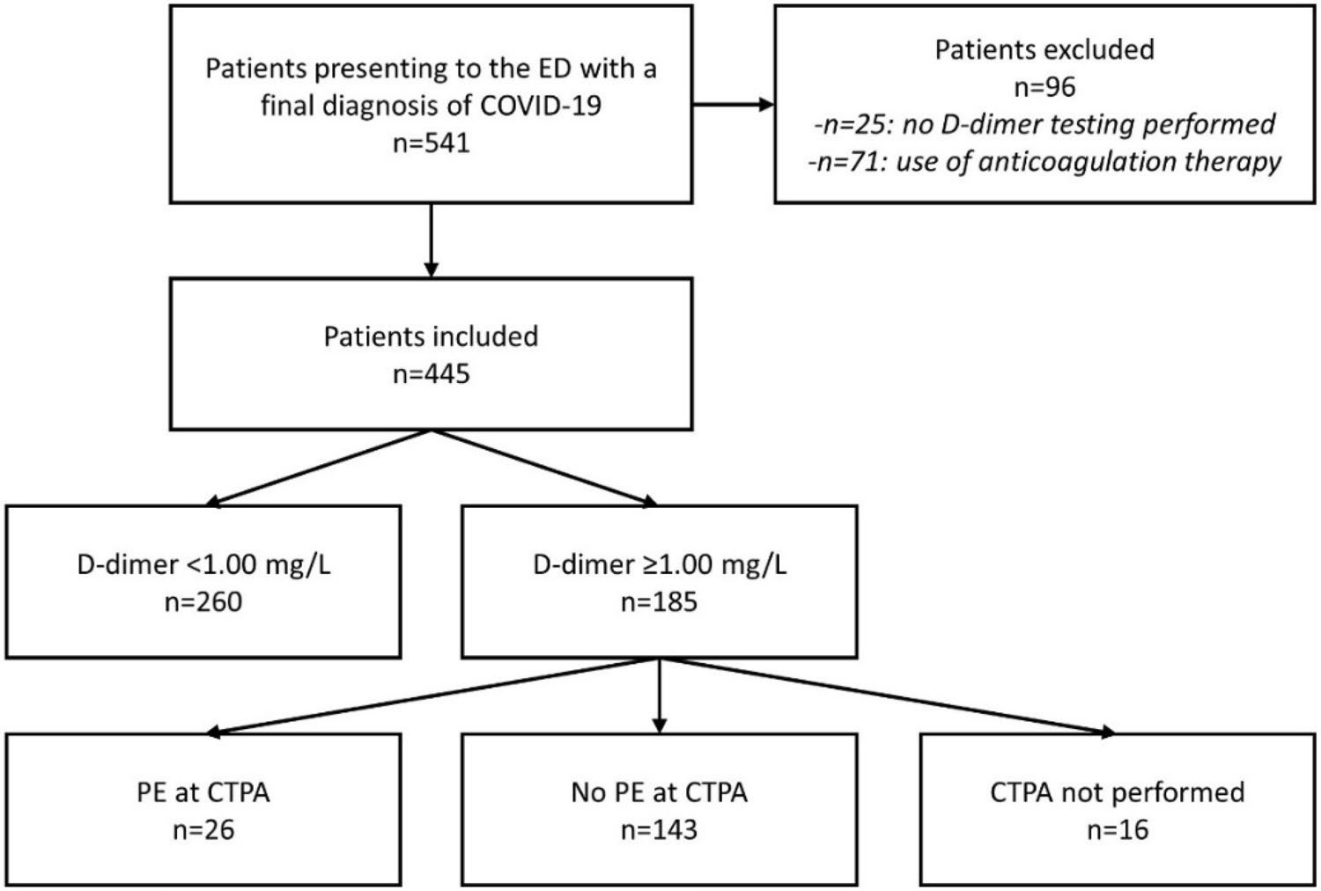
Flow of COVID-19 patients at the emergency department. *CTPA* CT pulmonary angiography, *ED* emergency department, *PE* pulmonary embolism

Figure 1 illustrates the flow of patients in the study. In the 3-month study period, 541 patients with a final diagnosis of COVID-19 presented to the ED. Of these, 25 patients (4.6%) were excluded because D-dimer testing was not performed in the ED (of whom 5 had CTPA in the ED, but none with PE), and 71 patients (13.1%) because they used anticoagulation therapy. Of 445 included patients, 258 (58.0%) presented to the Academic Medical Center and 187 (42.0%) to the VU University Medical Center, 183 (41.1%) were female, and median age was 63 years (IQR 52–73). Overall, 339 patients (76.2%) were admitted to the hospital, whereas 106 (23.8%) were discharged home from the ED.

**Fig. 2:**
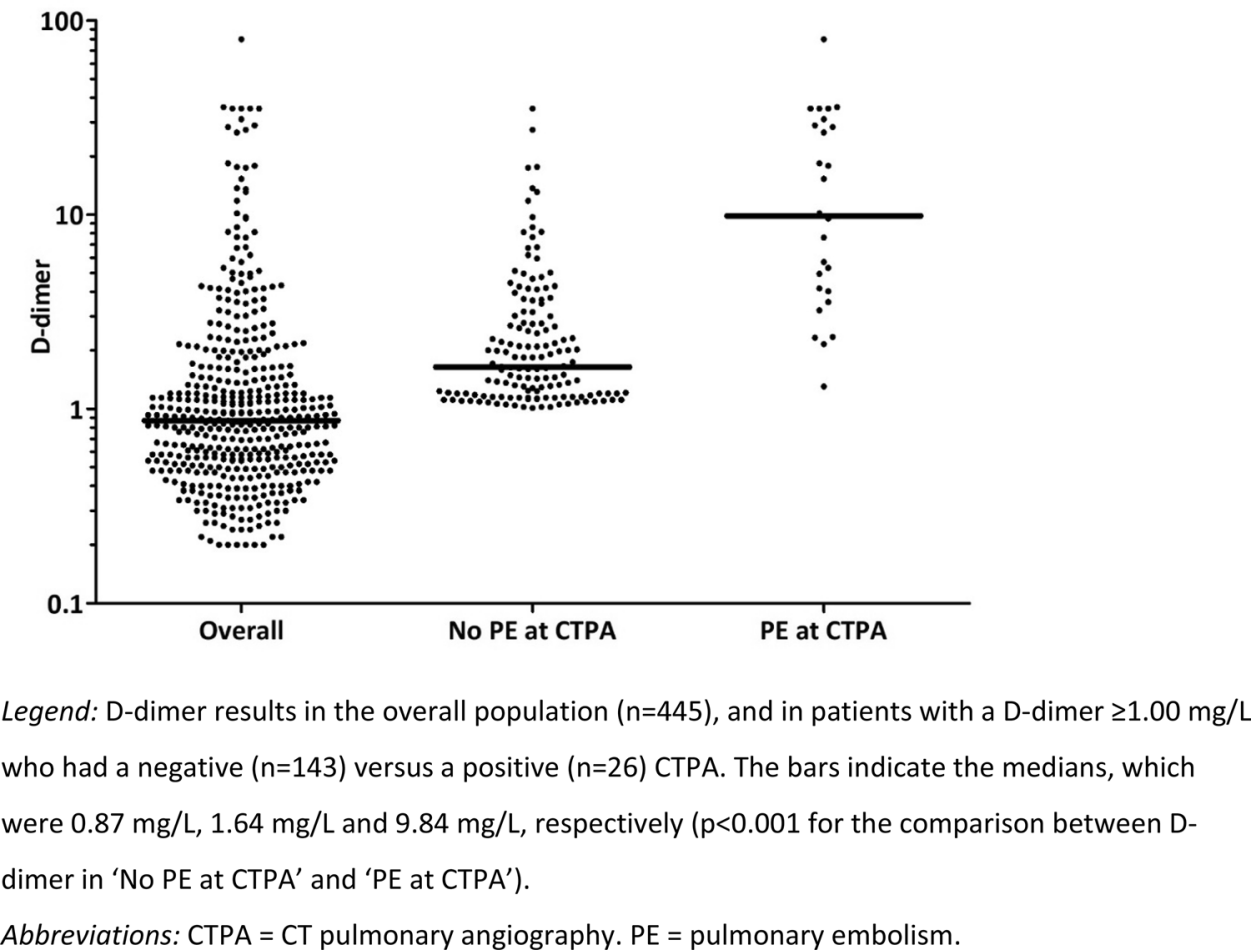
D-dimer results in COVID-19 patients at the emergency department

Outcomes of our screening algorithm for PE are summarized in Table 1, and D-dimer results in Fig. 2. In the overall study group, median D-dimer was 0.87 mg/L (IQR 0.52–1.57; range 0.20–80.00), and 185 patients (41.6%; 95%CI 37.0–46.3) had a D-dimer ≥ 1.00 mg/L. In line with the local protocol, CTPA was performed in 169 of these patients (91.4%), but the protocol was violated in 16 patients in whom CTPA was not performed because the attending physician considered the clinical suspicion of PE negligible (n = 10), because of inability of the patient to follow instructions (n = 2) or lie in supine position (n = 1), because of renal failure (n = 2), or because of initiation of palliative care (n = 1). CTPA showed PE in 26 of these 169 patients (15.4%; 95%CI 10.3–21.7), resulting in an overall PE detection rate in the complete study group of 5.8% (95%CI 3.9–8.4; n/N = 26/445) using this screening algorithm. The most proximal location of PE was central in 10 patients (38.5%), followed by segmental in 9 (34.6%), and subsegmental in 7 (26.9%). Median D-dimer was 9.84 mg/L (IQR 3.90–29.38; range 1.30–80.00) in the 26 patients with PE, and 1.64 (IQR 1.17–3.01; range 1.01–35.20) in the 143 without PE (p < 0.001; Fig. 2). PE prevalence increased with increasing D-dimer and ranged from 1.2% (95%CI 0.0–6.4) in patients with a D-dimer between 1.00 and 1.99 mg/L, to 48.6% (95%CI 31.4–66.0) in those with a D-dimer ≥ 5.00 mg/L (Table 1). Twenty-four patients underwent CTPA despite a D-dimer < 1.0 mg/L, of whom only 1 (4.2%; 95%CI 0.1–21.1) had PE (D-dimer in this patient was 0.67 mg/L and PE was segmental).

**Table 1:** Results of the screening algorithm for pulmonary embolism in COVID-19 patients at the emergency department

Overall study group		
*All patients*		n = 445
Median D-dimer (mg/L)		0.87 (IQR 0.52–1.57)
D-dimer ≥ 1.00 mg/L		n = 185 (41.6%; 95%CI 37.0–46.3)
CTPA performed in patients with D-dimer ≥ 1.00 mg/L		n = 169 (91.4%)
CTPA positive for PE		n = 26 (15.4%; 95%CI 10.3–21.7)

Subgroups based on admission status		
*Patients admitted to hospital after ED presentation*		n = 339
Median D-dimer (mg/L)		0.95 (IQR 0.58–1.85)
D-dimer ≥ 1.00 mg/L		n = 157 (46.3%; 95%CI 40.9–51.8)
CTPA performed in patients with D-dimer ≥ 1.00 mg/L		n = 146 (93.0%)
CTPA positive for PE		n = 24 (16.4%; 95%CI 10.8–23.5)
*Patients discharged home from the ED*		n = 106
Median D-dimer (mg/L)		0.55 (IQR 0.34–1.03)
D-dimer ≥ 1.00 mg/L		n = 28 (26.4%; 95%CI 18.3–35.9)
CTPA performed in patients with D-dimer ≥ 1.00 mg/L		n = 23 (82.1%)
CTPA positive for PE		n = 2 (8.7%; 95%CI 1.1–28.0)

CTPA outcomes by D-dimer results in patients with a D-dimer ≥ 1.00 mg/L*		
*D-dimer result (mg/L)*	*Total patients*	*Patients with PE at CTPA*
1.00–1.99	n = 85	n = 1 (1.2%; 95%CI 0.0–6.4)
2.00–2.99	n = 25	n = 3 (12.0%; 95%CI 2.5–31.2)
3.00–3.99	n = 12	n = 2 (16.7%; 95%CI 2.1–48.4)
4.00–4.99	n = 12	n = 3 (25.0%; 95%CI 5.5–57.2)
≥ 5.00	n = 35	n = 17 (48.6%; 95%CI 31.4–66.0)

*CTPA* CT pulmonary angiography, *ED* emergency department, *IQR* inter quartile range, *PE* pulmonary embolism

*Patients in whom CTPA was not performed (n = 16) were excluded from the analysis of CTPA outcomes by D-dimer results

In the subgroup of 339 patients admitted to hospital, 157 patients (46.3%; 95%CI 40.9–51.8) had a D-dimer ≥ 1.00 mg/L, of whom 146 underwent CTPA, which demonstrated PE in 24 (16.4%; 95%CI 10.8–23.5). This was approximately twice as high compared to the PE rate in the subgroup of 106 patients who were discharged home from the ED: 28 patients (26.4%; 95%CI 18.3–35.9) had a D-dimer ≥ 1.00 mg/L, and CTPA showed PE in 2 out of 23 (8.7%; 95%CI 1.1–28.0) in whom this was performed.

## Discussion

In EDs, deciding which COVID-19 patients should undergo CTPA for diagnosing PE is a major clinical challenge. The present study shows that, by routinely applying a screening algorithm consisting of D-dimer testing followed by CTPA if D-dimer is ≥ 1.00 mg/L, PE is frequently diagnosed in COVID-19 patients, which has important therapeutic consequences [14]. Our screening algorithm was based on a D-dimer threshold of 1.00 mg/L, which is used in various validated diagnostic algorithms for PE in the non-COVID-19 population, such as the YEARS algorithm [7, 8]. Because D-dimer testing is likely to be less specific in COVID-19 patients, we did not apply a clinical pretest probability to identify patients in whom a higher D-dimer threshold (1.00 versus 0.50 mg/L) can be used.

Several recent studies have suggested that an even higher D-dimer threshold can be used for selecting patients for CTPA in COVID-19 patients [15, 16]. However, these studies usually selected this threshold based on the optimal cutoff between sensitivity and specificity on the ROC curve, which tends to lead to suboptimal values for both of these, resulting in an unacceptable proportion of false negative D-dimer results and missed PEs [17]. Still, in the current analysis, only 1.2% (95%CI 0.0–6.4%) of COVID-19 patients with a D-dimer between 1.00 and 1.99 mg/L had PE, and not performing CTPA could be considered if confirmed in future prospective studies. However, prevalence of PE in patients with a D-dimer ≥ 2.0 was high, and CTPA should be performed.

An advantage of the screening algorithm applied in our study is that it is simple and straightforward: D-dimer testing is routinely done in every COVID-19 patient in the ED, and the decision to perform CTPA fully relies on the D-dimer result, regardless of clinical gestalt or the need to assess the presence of items from the YEARS algorithm or the Wells’ rule. Despite its retrospective design, a strength of our study is that the screening algorithm was prospectively implemented and systematically applied in a large group of consecutive COVID-19 patients presenting to the ED. The risk of selection bias was considered limited as protocol violations were infrequent: D-dimer was not performed in only 5.3% of COVID-19 patients, and CTPA was not performed in 8.6% of patients with a D-dimer ≥ 1.00 mg/L. By nature of the local protocol, some patients with a D-dimer < 1.00 mg/L may have had PE, as CTPA was not systematically performed in these patients. Therefore, we cannot draw conclusions about the safety of the algorithm, which could be the subject of future prospective management studies.
